# Characteristics and Effectiveness of Mobile- and Web-Based Tele-Emergency Consultation System between Rural and Urban Hospitals in South Korea: A National-Wide Observation Study

**DOI:** 10.3390/jcm12196252

**Published:** 2023-09-28

**Authors:** WooSung Choi, YongSu Lim, Tag Heo, SungMin Lee, Won Kim, Sang-Chul Kim, YeonWoo Kim, JaeHyuk Kim, Hyun Kim, HyungIl Kim, TaeHun Lee, Chol Kim

**Affiliations:** 1 Department of Emergency Medicine, Gachon University Gil Medical Center, Incheon 21565, Republic of Korea; choiwoosung@gilhospital.com; 2 Department of Emergency Medicine, Gachon University College of Medicine, Incheon 21565, Republic of Korea; 3 Department of Emergency Medicine, Chonnam National University Medical School, Gwangju 61469, Republic of Korea; dcheo@hanmail.net (T.H.); magicwizard2@hanmail.net (S.L.); 4 Department of Emergency Medicine, Cheju Halla General Hospital, Jeju 63127, Republic of Korea; wkim00@naver.com; 5 Department of Emergency Medicine, Chungbuk National University College of Medicine, Cheongju 28644, Republic of Korea; ooiarahan@nate.com; 6 Department of Emergency Medicine, Andong Medical Center, Andong 36743, Republic of Korea; intex@daum.net; 7 Department of Emergency Medicine, Mokpo Hangook Hospital, Mokpo 58643, Republic of Korea; aseptic@naver.com; 8 Department of Emergency Medicine, Yonsei University Wonju College of Medicine, Wonju 26426, Republic of Korea; khyun@yonsei.ac.kr; 9 Department of Emergency Medicine, Dankook University College of Medicine, Cheonan 31116, Republic of Korea; hilovesjj@naver.com; 10 Department of Emergency Medicine, Chuncheon Sacred Heart Hospital, Chuncheon 24253, Republic of Korea; ion2674@naver.com; 11 Department of Emergency Medicine, Saint Carollo General Hospital, Suncheon 57931, Republic of Korea; chol.carollo@gmail.com

**Keywords:** teleconsultation, telemedicine, mobile, transportation

## Abstract

(1) Background: The government of South Korea has established a nationwide web- and mobile-based emergency teleconsultation network by designating urban and rural hospitals. The purpose of this study is to analyze the characteristics and effectiveness of the tele-emergency system in South Korea. (2) Methods: Tele-emergency consultation cases from May 2015 to December 2018 were analyzed in the present study. The definition of a tele-emergency in the present study is an emergency consultation between doctors in rural and urban hospitals via a web- and mobile-based remote emergency consultation system (RECS). Consultations through an RECS are grouped into three categories: medical procedure or treatment guidance, image interpretation, and transportation requests. The present study analyzed the characteristics of the tele-emergency system and the reduction in unnecessary transportation (RUT). (3) Results: A total of 2604 cases were analyzed in the present study from 2985 tele-emergency consultation cases. A total of 381 cases were excluded for missing data. Consultations for image interpretation were the most common in trauma cases (71.3%), while transfer requests were the most common in non-trauma cases (50.3%). Trauma patients were more frequently admitted to rural hospitals or discharged and followed up with at rural hospitals (20.3% vs. 40.5%) after consultations. In terms of disease severity, non-severe cases were statistically higher in trauma cases (80.6% vs. 59.4%; *p* < 0.001). The RUT was statistically highly associated with trauma cases (60.8% vs. 42.8%; *p* < 0.001). In an analysis that categorized cases by region, a statistically higher proportion of transportation was used in island regions (69.9% vs. 49.5%; *p* < 0.003). More RUT was associated with non-island regions (30.1% vs. 50.5%; *p* = 0.001). (4) Conclusions: The tele-emergency system had a great role in reducing unnecessary patient transportation in non-severe trauma cases and non-island rural area emergency cases. Further research is needed for a cost/benefit analysis and clinical outcomes.

## 1. Introduction

Tele-emergency services are defined as remote-care medical services for acute illness, injury, and the acute exacerbation of chronic disease, including initial evaluations, diagnoses, treatments, the coordination of care, and disposition [1,2,3,4,5,6]. As technology advances and connectivity to internet networks increases, telemedicine and telehealth services have become more popular, but service availability and access system implementation are still globally challenging issues [7,8,9,10].

After the COVID-19 pandemic, medical and social environments have changed significantly. This worldwide outbreak led to a transformation in healthcare environments, from healthcare professionals’ clinical infection control protocols to whole emergency medical service systems [11,12,13,14]. These changes have resulted in a shift towards remote medical services, such as telemedicine and teleconsultation, rather than direct face-to-face interactions. Several studies have been conducted to analyze the various aspects of telemedicine and tele-consultation, including their methods, effectiveness, clinical outcomes, and user satisfaction [15,16,17,18,19,20].

Unlike chronic diseases, emergency diseases or injuries require rapid diagnoses and urgent management, which have a significant impact on patients’ clinical prognoses. In emergency medicine, it is critical to provide high-quality healthcare service to patients, without any interference from regional, environmental, or socioeconomic factors. Various methods have been attempted to provide medical services to vulnerable areas, and information-technology-based emergency teleconsultations between rural and urban hospitals have been developed and studied to provide emergency medical services to vulnerable areas as one solution [21,22,23,24,25,26,27,28]. In particular, mobile emergency telemedicine systems could help pre-hospital patient management, reduce mortality in cases in which transportation takes a long time, and expand standard healthcare services, especially in emergencies [29,30].

In South Korea, the Ministry of Health and Welfare has directed the step-wise development of the nationwide emergency teleconsultation network since 2015, designating urban and rural hospitals. Tele-emergency systems have been in operation to provide emergency medical services to vulnerable areas by using remote emergency consultation systems (RECSs). A RECS is a web- and mobile-based designed tele-emergency consultation system and provides high accessibility and portability. This property makes it highly suitable for emergency or critical consultation environments. Additionally, its cloud-based architecture provides an advantage for initial system setup, management, and maintenance, especially in rural regions.

There have been some previous studies analyzing the outcomes and benefits of teleconsultations of specific diseases or regions, but research on emergency consultations regardless of a specific disease, injury, or region with easily accessible and expandable and web- and mobile-based tele-emergency systems is lacking.

The present study aims to analyze the characteristics and effectiveness of nationwide web- and mobile-based tele-emergency consultation cases performed between rural and urban hospitals in South Korea.

## 2. Materials and Methods

### 2.1. Geographical and Healthcare Infrastructural Characteristics and Tele-Emergency Systems in South Korea

**Figure 1:**
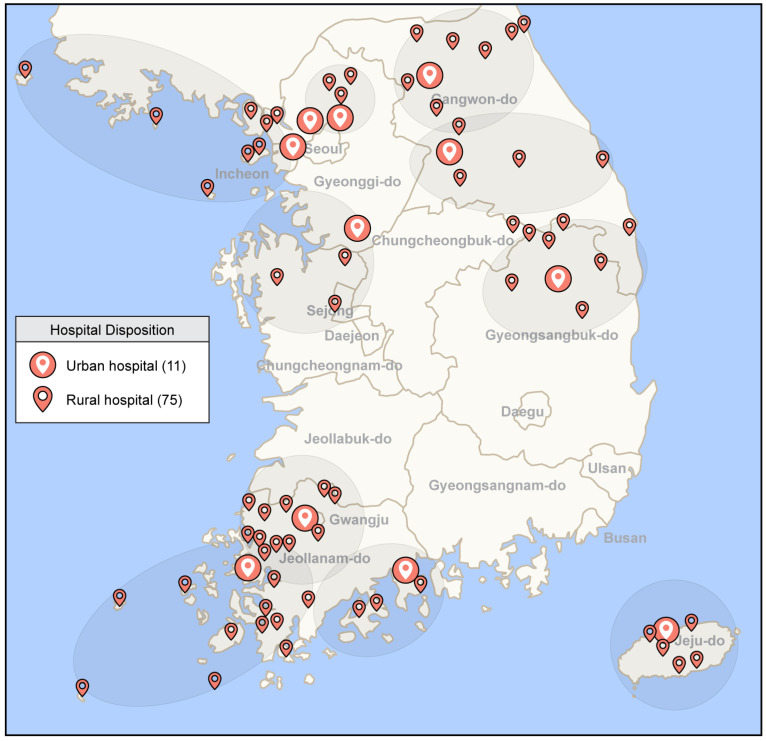
Tele-emergency network in South Korea.

South Korea is a peninsula surrounded by the sea on three sides, with 3170 islands of varying sizes [31]. The eastern side of the territory has several mountain ranges that create an isolated region on the inner side of the mountains. Over 50% of the entire population is concentrated in the capital and large metropolitan areas [32]. Various types of industrial infrastructure have been constructed focused on cities with a certain number of residents; the same is true for medical infrastructure. This trend of geopolitical characteristics makes it difficult for people to access healthcare services on islands or in remote mountain areas, especially emergency medical services [33]. To supplement the shortage of rural healthcare personnel, the South Korean government dispatches medical school graduates before residency as medical officers instead of conscription, providing primary medical care in rural areas. However, this often results in deploying doctors with limited clinical experience. To ensure that emergency medical services are provided to vulnerable areas, a RECS has been established step by step since 2015 by the government of South Korea, which designates urban and rural hospitals to provide a tele-emergency system. In 2021, the tele-emergency system was operational with 11 urban hospitals and 75 rural hospitals (Figure 1).

### 2.2. Development and Application of a Remote Emergency Consultation System (RECS)

**Figure 2:**
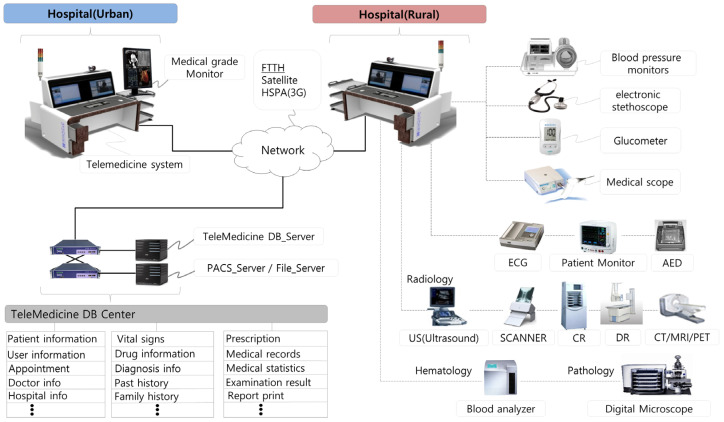
Diagram of the teleconsultation system configuration.

For emergency teleconsultations, a specific modality is needed for healthcare professionals at rural and urban hospitals for the management of patients’ medical status. A remote emergency consultation system (RECS) has been developed for video and voice transmissions and the sharing of patients’ medical status between healthcare professionals. The RECS is designed for particular functions, such as providing an electronic medical record (EMR) with cloud-based architecture. RECSs allow healthcare professionals to access a central server via the internet to send, store, and download patients’ clinical information, including web- and mobile-based picture archiving and communication systems (PACSs) with encrypted data transmission through SSL VPNs (Secure Sockets Layer Virtual Private Network) or IPsec VPNs (Internet Protocol Security Virtual Private Network). Patient clinical data that are collected from various diagnostic medical devices and transmitted via internet communication networks depend on region and network infrastructure. Urban and some rural areas mainly used fiber-to-the-home (FTTH) networks with speeds up to 10 Gbps, and in rural and island areas, long-term evolution (LTE) with speeds up to 100 Mbps or 3G (Third Generation) HSPA(High-Speed Packet Access) with a maximum of 14 Mbps are used. Satellite communication with a bandwidth of 1 Mbps to 2 Mbps is used in cases at sea or for patient transportation by boat (Figure 2). Healthcare professionals can participate in emergency teleconsultations by using the RECS application on their personal computers or cellphones to register patients’ medical status and manage their clinical data and medical images. The RECS provides an efficient environment for emergency teleconsultation through a video conference system and face-to-face or real-time on-scene sharing of patients’ clinical status.

### 2.3. Tele-Emergency Systems in South Korea: Definition and Application Process

The tele-emergency system in this study is defined as a medical service for emergency teleconsultations between doctors in a rural hospital and an urban hospital using the RECS. The urban hospital is mainly a tertiary hospital or regional emergency medical center designated by the government which has emergency teleconsultation capacity, including facility, equipment, and medical staff. The rural hospital is a medical institution or healthcare center located in a vulnerable area that has entered into a tele-emergency network participation agreement with an urban hospital and the government.

Healthcare professionals from hospitals designated on the tele-emergency network can access RECS version 2.0 (BIT COMPUTER, Seoul, South Korea) by visiting the RECS web page (www.recs.nemc.kr, accessed on 18 August 2023). Authorized approval from the National Emergency Medical Center under the Ministry of Health and Welfare is needed to access RECS. Users can download the mobile application of RECS (IOS version 4.03, Android version 3.29) to their smartphones from the RECS website. After logging into the RECS via web or mobile application, healthcare professionals can request or respond to consultations and can review patients’ clinical information.

In the consultation request process, tele-emergency consultations through RECS are defined into three categories: medical procedure guidance or treatment consultations, image interpretation consultations, and transportation consultations. The doctor requesting a consultation categorizes the reason for the consultation first and decides on one of three categories on the RECS consultation request page. Medical procedure guidance and treatment consultations include emergency medical procedure guidance, laboratory result interpretation, and recommendations for treatment plans. Image interpretation consultations include interpretation of X-rays, computed tomography, magnetic resonance images, or ultrasound images. Transportation consultations include discussions about and recommendations on transporting patients to urban hospitals and confirmation of acceptance of a patient’s transportation to an urban hospital. After the categorization of the reason for consultation, doctors in rural hospitals record the patient’s purpose for the consultation in detail by uploading clinical information, such as the patient’s history, laboratory data, images of the injured area, electrocardiogram (ECG), etc. Radiologic images are uploaded through Web a PACS connected to RECS.

**Figure 3:**
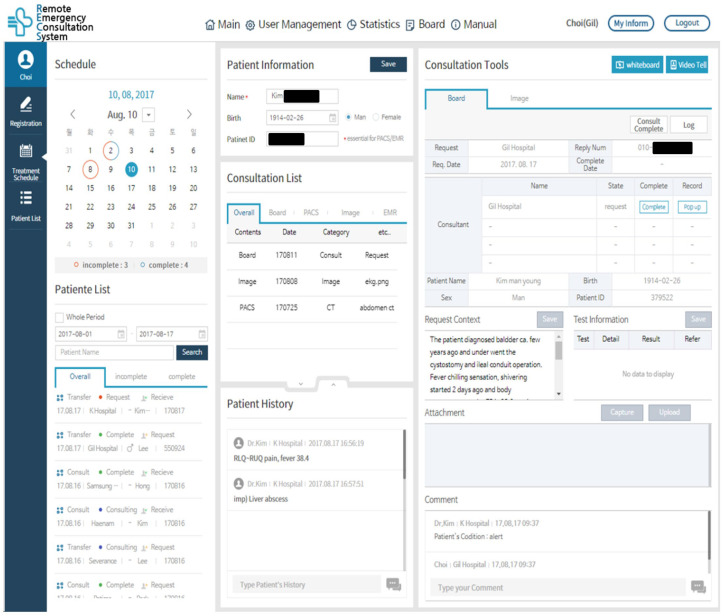
RECS (remote emergency consultation system) consultation response page on the website.

**Figure 4:**
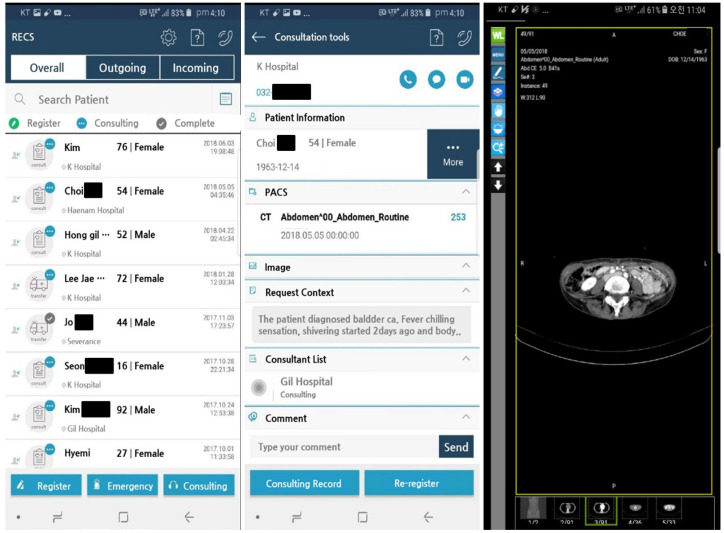
The mobile version of the RECS consultation page and a PACS image.

In the consultation response process, the doctor can access the page of the patient requestion the consultation by clicking on the patient list. On individual patient pages, urban hospital doctors can access uploaded patients’ clinical information medical data and radiologic images. Then, based on a patient’s clinical condition, they can record a consultation answer and recommendation appropriate to the reason for consultation. If a video consultation is needed, the doctor can initiate a video conference through the “video tell” option in the top right corner of the RECS page or the camera icon of the mobile application. The mobile application of the RECS is designed to have a user interface similar to the RECS web page (Figure 3 and Figure 4).

### 2.4. Study Design

The tele-emergency system was developed in 2015 and has been applied for emergency teleconsultations. This study analyzed cases between 11 urban hospitals and 75 rural hospitals from May 2015 to December 2018. Cases with missing critical data (e.g., the reason for the consultation, consultation result, or the trauma/non-trauma determination) were excluded. Enrolled cases were categorized into trauma vs. non-trauma and island vs. non-island to analyze differences in emergency teleconsultation characteristics (involving the region, time of consultation, consultation request method, reason for the consultation, consultation result, and reduction in unnecessary transportation). The definition of reduction in unnecessary transportation (RUT) case is if the consultation result is a patient’s admission to a rural hospital or discharge and follow-up monitoring from a rural hospital instead of transport to an urban hospital after emergency teleconsultation. This is due to the fact that, in general, rural hospitals make consultation requests for cases that are beyond their treatment or disposition capacity. Without tele-emergency consultations, these patients would be more likely to be transported to urban hospitals rather than being admitted or discharged from rural hospitals. Disease severity is categorized by the Korean Triage and Acuity Scale (KTAS). KTAS was developed based on the Canadian Triage and Acuity Scale (CTAS) and Japan Triage and Acuity Scale (JTAS) with consideration of characteristics of emergency medical service and medical infrastructure. KTAS categorizes a patient’s severity into five levels based on the patient’s subjective or objective signs and symptoms; the most severe patient is categorized as KTAS 1, while the least severe patient is categorized as KTAS 5 [34,35,36,37].

### 2.5. Statistical Analysis

Data were analyzed statistically using SPSS statics version 24.0 (SPSS Inc., Chicago, IL, USA). Univariate analysis for categorized variables (gender, time of consultation, consultation request method, reason for consultation, consultation result, disease severity, and reduction in unnecessary transportation) was performed using the chi-square test. The Student’s *t*-test was performed to analyze normally distributed continuous variables (age). Continuous variables were reported as mean and standard deviation. Differences with a *p*-value < 0.05 were considered to be statistically significant [38,39].

### 2.6. Ethical Statement

This study’s design was reviewed and approved by the institutional review board of Gachon University Gil Hospital (GCIRB2021-445). The requirement for informed consent was waived due to the retrospective nature of the study.

## 3. Results

**Figure 5:**
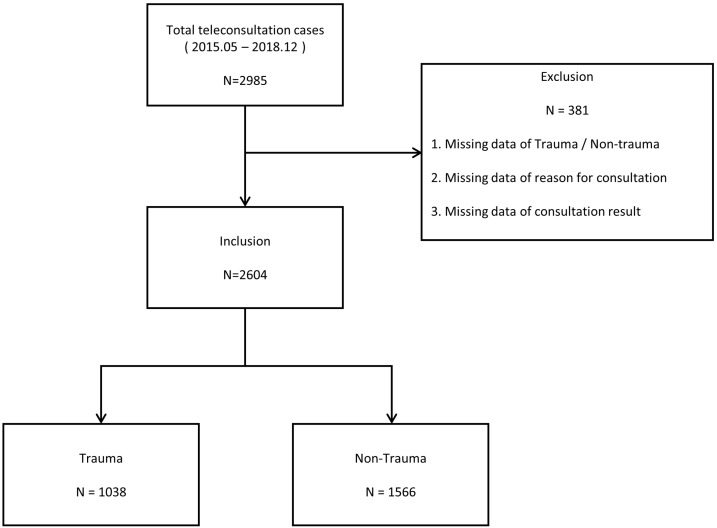
Flow chart of the study design.

Between May 2015 and December 2018, a total of 2985 tele-emergency consultations were performed through the RECS. A total of 381 cases were excluded due to missing data, and a total of 2604 cases were analyzed in the present study (Figure 5).

The basal characteristics of the comparison between trauma and non-trauma tele-emergency consultation cases are described in Table 1. Males were dominant in trauma cases (*n* = 684, 65.9%; *p* < 0.001), and the patients’ age was higher in non-trauma cases (56.0 ± 23.2 vs. 65.0 ± 20.9; *p* < 0.001). Tele-emergency consultations for trauma cases most often took place between 18:00 and 23:59 (*n* = 428, 41.2%), while non-trauma cases took place between 12:00 and 17:59 (*n* = 523, 33.4%). For both trauma and non-trauma cases, the most frequently used consultation method was a video consultation. The proportion of video consultation requests was higher for trauma cases (92.5% vs. 75.9%; *p* < 0.001). The reasons for consultation in the trauma cases and non-trauma cases showed significant statistical differences (*p* < 0.001). Image interpretation was the most common in trauma cases (*n* = 740, 71.3%), while transfer requests were the most common in non-trauma cases (*n* = 787, 50.3%). The consultation result also showed a significant statistical difference (*p* < 0.001). Trauma patients were more frequently admitted to rural hospitals or discharged and followed up with at rural hospitals (*n* = 211, 20.3%; *n* = 420, 40.5%), but non-trauma patients were transferred to urban hospitals more often (*n* = 896, 57.2%). In terms of disease severity, the non-severe cases (KATAS 3–5) were statistically higher in trauma cases (*n* = 785, 80.6% vs. *n* = 866, 59.4%; *p* < 0.001). The RUT was statistically highly associated with trauma cases (*n* = 631, 60.8% vs. *n* = 670, 42.8%; *p* < 0.001) (Table 1).

The characteristics of the comparison between island and non-island tele-emergency consultation cases are described in Table 2. There was no statistical difference in the time of consultation between island and non-island regions (*p* = 0.4). The reason for the tele-emergency consultation showed a significant statistical difference (*p* < 0.001). From the island, transfer requests were highly dominant (*n* = 45, 61.6%) while image interpretation was the most common in non-island regions (*N* = 1403, 55.4%). In both groups, the most common consultation result was transportation, but a statistically higher proportion of transportation was noted for island regions (*n* = 51, 69.9% vs. *n* = 1252, 49.5%; *p* < 0.003). In terms of disease severity, the non-severe cases (KATAS 3-5) were statistically higher in non-island regions (*n* = 31, 47.0% vs. *n* = 1620, 68.4%; *p* < 0.001). More RUT was associated with non-island regions (*n* = 22, 30.1% vs. *n* = 1279, 50.5%; *p* = 0.001) (Table 2).

## 4. Discussion

This study analyzed the characteristics and effectiveness of the nationwide web- and mobile-based tele-emergency consultation system in South Korea. The present study shows that for trauma cases, rural hospitals mostly requested image interpretation, and patients were more frequently admitted to or discharged from rural hospitals than transportated after tele-emergency consultations. This means tele-emergency consultations performed a significant role in reducing the unnecessary transportation of non-severe trauma patients. Island regions had a higher transportation rate with more severe cases than non-island regions, and the effect of RUT was higher in non-island regions.

Some prior studies have investigated the effect of telemedicine and teleconsultation. Armstrong et al. in their study of the experimental telemedical link between the Aberdeen Royal Hospital in Scotland and the Peterhead Community Hospital showed a 58% decrease in transportation (70 cases out of 116 cases) [40]. Lambrecht showed that telemedicine prevented unnecessary transportation for 53% of patients with minor injuries [41]. Natafgi et al. showed that 1175 transfers and USD 3823 were saved due to the Avera eMergency tele-emergency program (Sioux Falls, DS, USA) in 85 rural hospitals across seven states for 6 years [42]. The present study also shows a total of 631 cases (60.8%) were associated with an RUT, 211 patients were admitted to rural hospitals (20.3%), and 420 patients were discharged from rural hospitals (40.5%) in the total number of trauma consultation cases. Furthermore, our study also showed the effect of the RUT was significantly higher in trauma cases than in non-trauma cases (*n* = 631, 60.8% vs. *n* = 670, 42.8%; *p* < 0.001).

One of the most significant advantages of mobile devices (e.g., cellphones) is real-time direct on-scene sharing. Some prior studies have shown the effect of video tele-consultations, especially in trauma or injury cases. Roger et al. showed from a study of using telemedicine for real-time video consultations between a trauma center and community hospital that more than 80% of the referring providers felt that telemedicine consultations improved care [43]. Tsai et al. showed mobile camera phones are feasible for the remote management of extremity wounds, which is equivalent to 68% to 90% of diagnoses [44]. Our study also showed that the proportion of video consultation requests was especially higher in trauma cases (92.5% vs. 75.9%; *p* < 0.001). For trauma patients especially, visualized information of the injured site is critical. For the visualization of an injury site or the whole body regardless of the patient’s posture, position, or place, real-time video sharing is greatly advantageous. Furthermore, with real-time on-scene sharing, urban hospital doctors can also provide real-time guidance on medical procedures to rural hospital doctors [45]. This environment allows rural hospital doctors to perform critical emergency procedures for patients even if a rural hospital doctor does not have much experience with specific medical procedures.

The most notable characteristics of tele-emergency consultations in the present study were the significant differences in the reason for consultation, consultation request method, and consultation result between trauma and non-trauma cases, as well as the effect of the reduction in unnecessary transportation in both cases. More video consultation requests in trauma cases (*N* = 960, 92.5% vs. *N* = 1186, 75.9%) could be affected by the convenience of real-time on-scene sharing to visualize the site of injury. In trauma cases, tele-emergency consultation requests for image consultation are significantly more frequent (*N* = 740, 71.3% vs. *N* = 681, 43.5%), while for non-trauma cases, transport requests are more frequent (*N* = 269, 25.9% vs. *N* = 787, 50.3%). A possible reason for this is that image interpretation from computed tomography or X-rays has critical diagnostic value in trauma cases. However, in non-trauma cases, making a treatment plan for a patient and transferring them to an urban hospital for more appropriate and definitive treatment depending on the disease severity are the most critically important factors. Another characteristic of tele-emergency consultations was the higher transportation rate in island regions than in non-island regions. This could be due to the fact that the only methods of transfer from island regions are marine vessels or air transportation. The limited number of vessels and operation times may have led to more aggressive decisions around patient transportation. As a result, the effect on the RUT was higher in non-island regions than in island regions.

As the most important advantage of mobile and web PACSs, EMRs provide detailed information on a patient’s medical status to doctors and are used to make appropriate clinical decisions about patient management and adequate transportation. They can improve the quality of emergency treatments initially provided to patients and are helpful to prevent unnecessary transportation costs for non-emergency patients. The other advantage of the RECS is the reduced cost of the tele-emergency system set up at urban and rural hospitals. The setup, management, maintenance, and repair of teleconsultation system devices are highly cost-effective projects. Especially in rural hospitals on islands or in mountainous regions, their physical distance complicates system maintenance and management; as a result, system-related feedback between the user and developer cannot be immediate. These drawbacks could be overcome significantly by using a web- and mobile-based RECS.

Generally, in trauma cases, after tele-emergency consultations, the non-severe cases were admitted to rural hospitals for treatment, which had a positive effect on the RUT. In contrast, in severe cases, appropriate initial management and quick decisions could be implemented through tele-emergency consultations. Furthermore, urban hospital medical staff members can obtain information about a patient’s medical status in advance, allowing them to prepare for the planned operation or procedure. This environment is expected to bring significant benefits in terms of patient treatment.

### Limitations

The present study does have some limitations. Firstly, the RUT could not perfectly reflect the actual reduction in unnecessary transportation. Without the tele-emergency consultation system, the clinical decision in a large portion of these cases would have been to transfer the patient to an urban hospital, but not every decision would have been in favor of urban hospital transportation. For an accurate analysis of the RUT, additional research is needed to investigate changes in physicians’ clinical decisions and rural hospital treatment results before and after tele-emergency consultations. Secondly, our results suggest that tele-emergency consultations can reduce unnecessary transportation and contribute to the efficient use of medical resources. However, an accurate cost-effectiveness analysis was not conducted to determine the actual economic benefit, which is essential for evaluating the RECS. Further studies on the cost-effectiveness of tele-emergency cases are needed. Thirdly, the present study could not analyze the treatment outcomes of the tele-emergency consultation cases. One significant advantage of the RECS is that it provides an early-stage treatment guide, early transportation decisions, and clinical data on emergency patients in advance. Further studies for the evaluation of the long-term medical outcomes of patients for whom an RECS was used are needed.

## 5. Conclusions

Teleconsultation between rural and urban hospitals is expected to supplement the deficiency in medical services in rural areas. In South Korea, the web- and mobile-based tele-emergency system between rural and urban hospitals greatly reduced unnecessary patient transportation in non-severe trauma and non-island rural area emergency cases. Further research including an analysis of the cost-saving effect of the RUT and the impact of tele-emergency consultations on patients’ clinical outcomes are needed for a reliability assessment of the tele-emergency consultation system.

## Figures and Tables

**Table 1:** Basal Characteristics of Tele-emergency Consultations.

Characteristics		Total		Trauma		Non-Trauma		*p*-Value
		*N*	%	*N*	%	*N*	%	
Total		2604	100.0	1038	100.0	1566	100.0	
Gender								<0.001
	Male	1572	60.4	684	65.9	888	56.7	
	Female	1032	39.6	354	34.1	678	43.3	
Age (Years)								<0.001
Mean ± SD		61.0 ± 22.4		56.0 ± 23.2		65.0 ± 20.9		
Region		2604		1038		1566		0.320
	Island	73	2.8	25	2.4	48	3.1	
	Non-Island	2531	97.2	1013	97.6	1518	96.9	
Time of consultation		2604		1038		1566		<0.001
	00:00–05:59	339	13.0	137	13.2	202	12.9	
	06:00–11:59	506	19.4	148	14.3	358	22.9	
	12:00–17:59	848	32.6	325	31.3	523	33.4	
	18:00–23:59	911	35.0	428	41.2	483	30.8	
Consultation request method		2600		1038		1562		<0.001
	Video	2146	82.5	960	92.5	1186	75.9	
	Telephone	454	17.5	78	7.5	376	24.1	
Reason for Consultation		2604		1038		1566		<0.001
	Procedure and Treatment Guidance	127	4.9	29	2.8	98	6.3	
	Image interpretation	1421	54.6	740	71.3	681	43.5	
	Transfer request	1056	40.6	269	25.9	787	50.3	
Consultation Result		2604		1038		1566		<0.001
	Transportation	1303	50.0	407	39.2	896	57.2	
	Admission to rural hospital	553	21.2	211	20.3	342	21.8	
	Discharge from rural hospital	784	28.7	420	40.5	328	20.9	
Disease Severity		2433		973		1460		<0.001
	KTAS * 1–2 (severe)	782	32.1	188	19.3	594	40.7	
	KTAS * 3–5 (mild to moderate)	1651	67.8	785	80.6	866	59.4	
Reduction in Unnecessary Transportation (RUT) *		1301	50.0	631	60.8	670	42.8	<0.001

Note: KTAS *, Korean Triage and Acuity Scale; RUT *, Reduction in unnecessary transportation; Non-transportation cases are shown as a percentage of total teleconsultation cases.

**Table 2:** Comparison of characteristics between islands and non-islands.

Characteristics		Total		Island		Non-Island		*p*-Value
		*N*	%	*N*	%	*N*	%	
Total		2604	100.0	73	100.0	2531	100.0	
Time of consultation		2604		73		2531		0.400
	00:00–05:59	339	13.0	10	13.7	329	13.0	
	06:00–11:59	506	19.4	19	26.0	487	19.2	
	12:00–17:59	848	32.6	24	32.9	824	32.6	
	18:00–23:59	911	35.0	20	27.4	891	35.2	
Reason for Consultation		2604		73		2531		<0.001
	Procedure and Treatment Guidance	127	4.9	10	13.7	117	4.6	
	Image interpretation	1421	54.6	18	24.7	1403	55.4	
	Transfer request	1056	40.6	45	61.6	1011	39.9	
Consultation Result		2604		73		2531		0.003
	Transportation	1303	50.0	51	69.9	1252	49.5	
	Admission to rural hospital	553	21.2	10	13.7	543	21.5	
	Discharge from rural hospital	784	28.7	12	16.4	736	29.1	
Disease Severity		2433		66		2367		<0.001
	KTAS * 1–2 (severe)	782	32.1	35	53.0	747	31.6	
	KTAS * 3–5 (mild to moderate)	1651	67.8	31	47.0	1620	68.4	
Reduction in Unnecessary Transportation (RUT) *		1301	50.0	22	30.1	1279	50.5	0.001

Note: KTAS *, Korean Triage and Acuity Scale; RUT *, Reduction in unnecessary transportation; Non-transportation cases are shown as a percentage of total teleconsultation cases.

## Data Availability

The datasets generated and analyzed during the current study are not publicly available since they contain potentially identificatory information for each patient; however, they are available from the corresponding author upon reasonable request.
